# Psychological pathways of intercultural adaptation among international students in Europe: identity negotiation and cognitive dissonance in counselling and mentoring contexts

**DOI:** 10.3389/fsoc.2026.1894672

**Published:** 2026-10-22

**Authors:** Rita Takacs, Viktória Nagy, Zsuzsa Kaló, Zoltán Horváth

**Affiliations:** 1 Faculty of Informatics, ELTE Eötvös Loránd University, Budapest, Hungary; 2 Doctoral School of Informatics, ELTE Eötvös Loránd University, Budapest, Hungary; 3 Institute of Psychology, ELTE Eötvös Loránd University, Budapest, Hungary

**Keywords:** case studies, counselling psychology, intercultural adaptation, mentorship, university environment

## Abstract

**Introduction:**

Intercultural adaptation is a very essential aspect of psychological integration. As one of Europe’s leading universities, Eötvös Loránd University Budapest welcomes new groups of students every year. To help them navigate the university system and to support the cultural integration process, we organise mentoring programs for them. Our students come from every continent, with the majority hailing from countries in Africa, the MENA region, and Asia. The purpose of this study is to explore the challenges and processes related to the intercultural adaptation of international students, with a particular focus on identity reconstruction, coping strategies, and the role of mentoring and psychological support in facilitating successful integration.

**Methods:**

In this article, we will discuss two cases: the first case study revolves around an individual case, a student who, during this process, an individual participates in a series of counselling sessions, is seeking a new identity appropriate to their new situation, and the process of redefining identity takes place during a psychological consultation. The second case study revolves around group mentoring sessions: we summarise how international students perceive the university’s internal rules, how they manage their own adaptation accordingly, and what strategies they employ at different levels of coping. We also examine how their perceived reality aligns with their previous educational experiences in their home country and how they manage this cognitive dissonance.

**Results:**

The case studies highlight that intercultural adaptation involves a continuous process of reconciling prior educational and cultural experiences with the expectations of the host institution. Both cases underscore the importance of identity reconstruction and adaptive coping strategies in promoting successful integration. Students interpret the differences between their previous educational environments and university norms in their own unique ways; overcoming cognitive dissonance is key to adaptation.

**Discussion:**

Ultimately, we would like to draw some general conclusions for mental health counselling in higher education regarding which mentoring tools are most helpful and raise awareness of various topics among international students to facilitate their own integration. The results show that targeted mentoring tools and services that encourage structured self-reflection significantly facilitate international students’ adjustment. Raising awareness of cultural differences, academic expectations, and coping resources can further promote psychological well-being and successful integration into the university community.

## 1 Introduction

The Faculty of Informatics at Eötvös Loránd University Budapest (ELTE) has offered student counselling in English since 2014. To help the growing number of international students integrate each academic year, the Faculty of Informatics has implemented a multi-step approach to facilitate student integration and support their academic success (Nagy et al., 2025). The institution currently has nearly 1,200 international students, and that number is growing year by year.

In the Science, Technology, Engineering and Mathematics (STEM) field of higher education, a degree in Computer Science is among the most challenging; there is a significant dropout rate at both the B. S. and M. S. levels, and institutions employ various strategies to prevent dropout and retain students (Baker and Siryk, 1984).

Cultural adaptation raises significant questions for international students about how to address it in a university setting. The presence of soft skills that influence students’ adaptation is crucial: e.g., flexibility, adaptability, communication, and self-awareness (Genkova and Schreiber, 2025; Nguyen et al., 2026). The presence and prioritization of these skills are crucial for university integration and cultural adaptation. Academic success is also linked to cultural integration: students who successfully interpret their environment can achieve better academic results, which further strengthens their motivation (Bethel et al., 2020; Lai et al., 2023).

Likely, a student who perceives their daily struggles as failures and finds themselves facing situations day after day that contradict their previous beliefs may develop academic underachievement and subsequently lose their motivation to study (Mitchell and Paras, 2018; Owusu, 2025). Students often seek counselling or their mentor teacher, saying they may lack clarity regarding their role and expected activities and would prefer a different career, which can be a symptom of an unsuccessful coping mechanism (Boekestijn, 1988; Orbe, 2008).

Models of cultural adaptation describe this complex process as a multidimensional one, encompassing cognitive, affective, and behavioural components (Genkova and Schreiber, 2025; Genkova et al., 2025). In this sense, intercultural competence refers not only to knowledge but also to the tolerance of ambiguity and the ability to manage contradictions.

1. There are several crucial stages in the development of identity. One of the most important levels is identity commitment, which reflects the extent to which an individual is able to regard certain values, goals, or roles as their own (Marcia, 1966).
2. The closure of individuation is when the individual stops weighing alternatives and makes a final decision (Luyckx et al., 2006).

During accommodation, individuals are able to integrate new experiences and modify their existing identity structures accordingly (Piaget, 1968). Identity formation is a flexible and coherent process. Identity is flexible to the extent where its parts are activated differently in various contexts (McAdams, 1996; Janík, 2017). Cultural integration or adaptation is a very long process, and experiences of success and failure are ongoing (Bennett and Bennett, 2004).

It follows that the development of identity is both a flexible and context-dependent process; for this reason, it is critical to examine and analyse the specific environmental influences within which the simultaneous and mutually reinforcing processes of identity formation and adaptation take place. The transition to higher education is also the initial stage of becoming an adult, so it can encompass a period of multiple critical junctures during which young adults are confronted with academic, social, and cultural expectations. Although previous research has examined identity formation and university adjustment separately, little attention has been paid to how identity processes interact with contextual factors in the higher education environment in an international context. Understanding this relationship is particularly essential in increasingly international university environments, where students must continually reinterpret their self-concept while adapting to the expectations and communication channels of their new environment.

The higher education environment, particularly the Bologna education system (European Ministers of Education, 1999), contributes significantly to an individual’s ability to shape their own identity in response to the environment and to adapt. During university adaptation, individuals must adjust not only to academic requirements but also to institutional culture and the new social environment (Baker and Siryk, 1984). This process is closely linked to the dynamic development of identity, where commitment and accommodation may occur simultaneously (Qumseya, 2018; Wang and Huang, 2026).

Communication patterns also influence the processes of identity formation and adaptation. Most European cultures follow a linear communication style, which emphasises logical, goal-oriented, and structured information transfer (Hall, 1976). In contrast, in many other cultures—such as Asian or African contexts—circular or context-dependent communication is observed, where meaning unfolds more implicitly through relational and situational factors (Nisbett, 2003). These differences are also evident in the university setting and can influence students’ learning strategies as well as the activation of different aspects of their identity (Irrmann, 2005; Jade et al., 2026).

## 2 Theoretical background

The phenomena of conflicting cognitions is when the system of rules corresponding to their internal world does not match the expected outcome experienced in external reality (Festinger, 1957). According to the theory of cognitive dissonance, when an individual experiences two or more conflicting cognitions then the individual experiences discomfort and must reconcile the newly experienced system of norms with the old one (Festinger, 1957).

If cognitive dissonance cannot be resolved, defence mechanisms or maladaptive behavioural patterns may emerge, which can even result in avoidance behaviour. However, if support is provided at an appropriate level and processing begins, the result may be cognitive restructuring and an adaptive change in attitude.

### 2.1 Conceptual model: cultural difference - cognitive dissonance - identity negotiation - adaptation outcome

The proposed conceptual model, which posits a connection between Cultural Difference–Cognitive Dissonance – Identity Negotiation and Adaptive Outcome, is based on the premise that cultural differences experienced in a new educational environment can challenge students’ existing beliefs, values, and communication patterns, resulting in cognitive dissonance (Festinger, 1957). This psychological tension prompts individuals to reevaluate their self-concept and social roles, leading to a process of identity negotiation in which cultural, personal, and relational identities are activated, and adaptation to the demands of the new environment begins—that is, coping with cognitive dissonance (Ting-Toomey, 1999, 2015). Identity negotiation is particularly evident when an individual enters a different cultural environment, where they must fulfil the role of a student and strike a balance between maintaining a sense of self-continuity and adapting to new norms and expectations. Successfully managing tensions related to identity reconciliation facilitates psychological and sociocultural adaptation, whereas unresolved dissonance, on the contrary, leads to difficulties in adaptation, a sense of uncertainty, and a perceived decline in psychological well-being. Thus, adaptation can be understood as the result of a dynamic process in which cultural differences trigger cognitive dissonance, identity negotiation serves as a mediating mechanism, and the resulting identity transformation shapes the quality and outcome of adaptation (Ward and Szabó, 2023; Liu, 2017) (Figure 1).

**Figure 1:**
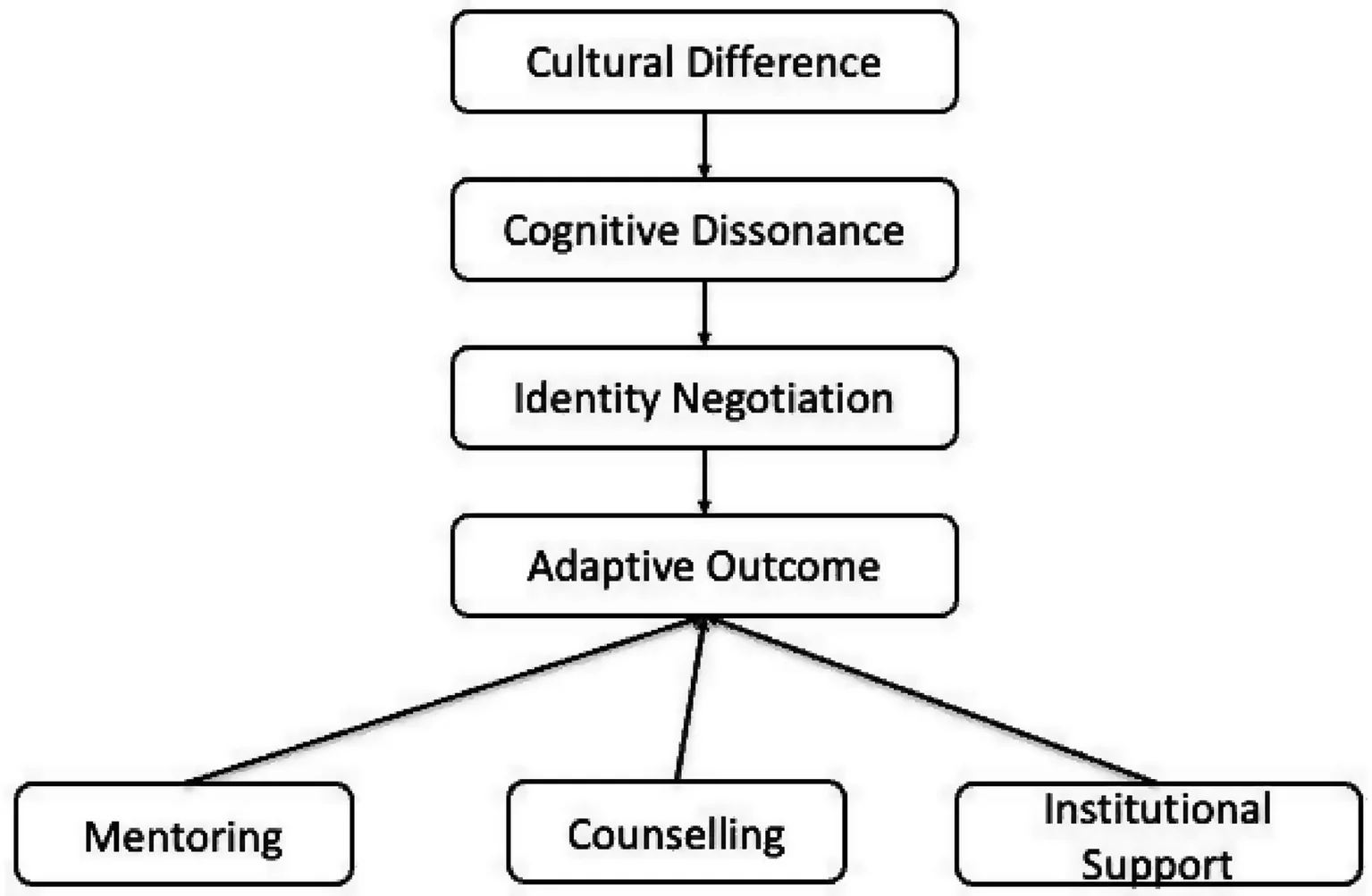
Conceptual framework.

In higher education, intercultural adaptation is increasingly described as a complex, multidimensional process, as we expect not only behavioural adaptation from the individual but also profound cognitive and emotional reorganisation in accordance with the new cultural environment (Bethel et al., 2020; Lai et al., 2023; Crisp, 2010).

This means that international students bear a significant responsibility, as they must navigate the discrepancies between their internalised cultural schemas and the normative expectations of the host academic environment. These discrepancies often lead to cognitive dissonance, which, from a psychological perspective, is a highly stressful state (Hoshino-Browne et al., 2005; Kokkoris and Kühnen, 2013).

Research published in the journal *Frontiers in Psychology* emphasises that international students must simultaneously navigate cultural maintenance and participation in the host culture, often amid significant cultural distance and institutional pressure (Bethel et al., 2020; Hofhuis et al., 2023). This dual demand creates structurally embedded contradictions: students are expected to preserve certain aspects of their identity while also internalising new norms (Boekestijn, 1988; Nguyen et al., 2026). The institution urges their adaptation, yet this is a prolonged process because in many cases these norms may be incompatible with prior institutional or societal socialization (Jade et al., 2026).

According to cognitive dissonance theory, individuals are motivated to reduce contradictions between different cognitions they have experienced to restore psychological equilibrium (Hoshino-Browne et al., 2005). In an intercultural context, however, these new cognitive experiences do not occur randomly but at a systemic level, placing significant responsibility on the individual (Mitchell and Paras, 2018; Owusu, 2025).

Empirical findings suggest that these tensions are particularly pronounced in the lives of college students, as they must adapt not only to the academic setting but also to the broader social environment (Baker and Siryk, 1984; Bethel et al., 2020), where differences in communication styles, expectations regarding autonomy, and grading systems create what is known as “culture shock” (Hall, 1976; Nisbett, 2003). For example, in the new educational system, students must reconcile often contradictory norms regarding authority, classroom participation, study habits, and academic requirements. These conflicts are not merely situational but often call into question fundamental aspects of self-concept, thereby intensifying dissonance and increasing the risk of maladaptive coping (Janík, 2017; Qumseya, 2018).

The literature highlights that the outcomes of adaptation depend not only on exposure to cultural differences, but also on the extent to which and how individuals process and integrate new content on a cognitive level (Genkova and Schreiber, 2025; Hofhuis et al., 2023). Studies show that unresolved tensions between home and host cultural orientations can, in most cases, negatively affect psychological well-being, while successful integration is associated with higher levels of adaptation and life satisfaction (Bethel et al., 2020; Lai et al., 2023). This is consistent with the theory that cognitive flexibility and meaning making contribute significantly to effective intercultural adaptation (Mitchell and Paras, 2018).

The work of Genkova and Schreiber (2025) further expands this academic approach by defining intercultural competence as a structured set of cognitive, affective, and behavioural abilities that help individuals navigate a new cultural environment more easily. Intercultural competence encompasses not only knowledge and skills but also the ability to tolerate ambiguity, reconcile conflicting norms, and regulate internal psychological tension (Genkova et al., 2025). Within this interpretive framework, cognitive dissonance is not merely a challenge but a developmental trigger that acts as a catalyst for the acquisition of competence.

From a European perspective, intercultural encounters often give rise to implicit assumptions, normative ambiguities, and value conflicts that require continuous cognitive calibration (Genkova et al., 2025; Irrmann, 2005). These findings reinforce the notion that dissonance manifests not only in overt cultural differences but also in discrepancies in expectations, more subtle communication patterns, and social evaluations (Hall, 1976; Nisbett, 2003).

To sum up, cognitive dissonance can be viewed as a central explanatory construct that helps us understand intercultural adaptation in higher education. It operates at the intersection of the following processes: acculturation processes (balancing aspects of the home and host cultures) (Boekestijn, 1988), identity negotiation (maintaining self-coherence) (Orbe, 2008; Karanikola and Panagiotopoulos, 2025), and adapting to situational demands in line with academic and social expectations (Baker and Siryk, 1984).

Within this interpretive framework, the resolution of dissonance—or its maladaptive management—becomes a key factor in both psychological well-being and academic persistence (Hofhuis et al., 2023). Throughout their time as students, conflicts arise between the expectations of the host university environment and the students’ own value systems and norms, often leading to cognitive dissonance. A clear clash of values can be observed between the values stemming from students’ collectivist culture (e.g., respect for hierarchy, indirect communication, prioritizing relational harmony) and the individualistic, autonomy-oriented norms of the European higher education environment (Erez and Earley, 1993).

The reconciliation of these values, known as value incongruence, manifests as an internal psychological conflict that takes an emotional toll on the individual, can lead to a lack of self-confidence, and may result in a form of behavioural inhibition (Schwartz, 1992). The students’ adaptive schemas, which they have applied successfully in the appropriate cultural environment thus far, must now be reinterpreted in this new context to adapt.

The counselling process is typically structured into several phases, including an initial stage focused on building rapport and clarifying the client’s concerns, followed by a goal-setting phase where the key issues and desired outcomes are identified. In the working phase, the counsellor and client collaboratively develop and apply strategies for change, while the final stage involves evaluation, integration of insights, and closure of the process (Sutton, 2021).

Narratives may also take the form of an internal monologue: through “inner speech” (its vocalization), individuals interpret their own feelings and actions, which supports self-reflection and the integration of experiences (Vygotsky, 1978).

Overall, it can be said that commitment to identity and accommodation are complex, dynamic processes. The university system, the challenges of continuous adaptation, and cultural-communication differences all contribute to the development of individuals’ identities from multiple directions. It is the individual’s task to continuously analyse the various identity structures and adapt to the changing environment (Crisp, 2010; Hofhuis et al., 2023). Existing studies have examined intercultural adaptation, but few studies have explored identity negotiation and cognitive dissonance simultaneously within counselling and mentoring settings.

The Importance of Psychological Adaptation

- In this article, we aim to explore micro-level processes that can help professionals supporting international student integration from various roles, such as psychologist, soft skill trainer pr mentor on how to facilitate adaptation at the individual or group level, as well as identify typical areas of concern in the university setting that require our attention.

Research question:

- What psychological mechanisms facilitate or hinder cultural adaptation in Europe for international students in higher education?

## 3 Methodology

### 3.1 Participant

Demographic data (We present a pseudo-identity constructed from a pattern observed across multiple individual counselling cases.) Since 2019, the proportion of international students in European higher education institutions has increased exponentially, with many of these students arriving from regions outside Europe, including the MENA region, Africa, and Asia. At the same time, the level of psychoeducation and trust toward institutions within this population has steadily grown. As a result, an increasing number of students have sought counselling services, while the number of crisis cases has risen sharply since 2023.

### 3.2 Background of the pseudo-cases

The Student Support Centre at the Faculty of Informatics of Eötvös Loránd University has been providing psychological counselling to international students since 2014. On average, approximately 100 university students utilize individual counselling services each academic year, while about 300 first-year students participate in mentoring programs annually. Both case studies present pseudo-cases, a decision primarily motivated by the institution’s ethical guidelines and data protection requirements; but we also wanted to present representative and theoretically informative examples that accurately reflect the common challenges experienced by international students during their adaptation process, thereby providing a useful resource for a wider range of professionals working in this field.

The cases presented in this study maintain professional credibility and originality, as they stem from this broad professional experience. The cases presented are not based on a predefined case study database, but rather on practical experience gained in the context of higher education counselling and mentoring. The first case is based on patterns repeatedly observed during individual counselling sessions. The second case stems from a group counselling context and represents the integration of approximately 10 similar cases, highlighting recurring themes, questions, and reactions related to adaptation and identity identified among the participants. To ensure strict compliance with data protection and confidentiality requirements, we did not directly disclose any individual student’s counselling files or notes. Instead, we compiled the cases as pseudo-cases (composite cases), combining characteristics, experiences, and challenges observed across multiple individuals, while completely masking their origins and other identifying information. This approach prevents the identification of any specific student while preserving the authenticity and educational value of the observed phenomena. We synthesised the relevant elements of several comparable cases into coherent narratives, thereby illustrating the theoretical concepts discussed in the article and linking them to practical examples.

The two case studies offer complementary viewpoints on the process of counseling and adaptation. The first case study is an in-depth examination of the student’s specific experiences, difficulties, and coping mechanisms during an individual counseling procedure. The second case study, on the other hand, focuses on interpersonal dynamics, shared experiences, and the significance of peer contact in the process of adaptation in a group counseling situation. The two scenarios complement one another and offer a more thorough picture of how psychological and cultural adaptation may be helped in various counseling contexts by looking at both individual and group-level processes.

#### 3.2.1 Method of data selection

The current dataset contains a total of 20 cases. We maintain the data in an Excel database, where we systematically classify it and record the frequency and occurrence of the cases.

#### 3.2.2 Definition of topics

The cases were selected on the basis of their relevance to the research topic, with particular attention paid to situations related to the crisis; we highlighted those recurring patterns which are key to the topic and which provided a basis for drawing conclusions. We paid particular attention to trends emerging over time, such as the increasing number of crisis-related cases observed year on year.

#### 3.2.3 The process of combining multiple cases into a pseudo-case

We constructed the pseudo-case by combining the common characteristics, recurring themes and shared experiences identified across the 20 cases. A pseudo-case does not represent a single specific case, but reflects the themes and patterns that occur most frequently in the data set, thereby creating a composite example that illustrates the broader trends observed during the analysis.

#### 3.2.4 Validity of the interpretation

Conclusions were drawn exclusively from recurring themes that appeared consistently across multiple cases, which helped to ensure that the interpretation reflects broader trends within the dataset.

The pseudo-cases were constructed through iterative qualitative synthesis of counseling notes and mentoring observations. Recurring themes related to identity negotiation, cognitive dissonance, and adaptation were identified through repeated discussions among the authors until conceptual agreement was reached.

## 4 Psychological process 1: identity negotiation

Through the narrative development of this pseudo-identity, we illustrate the individual experiences and developmental trajectory of an international student within the counselling process.

- 20–25-year old student enrolled to a BSc program in STEM.
- Country of origin, context of residence in Europe: Originally from the Middle-East North Africa region (MENA).
- Context: Counselling/mentoring framework. The student signed up for an individual counselling process.

### 4.1 Context and participant

The first case involves a 20-25-year old student who came to seek one-on-one counselling at the Student Support Centre after spending 6 months in Hungary. During the weekly mentoring sessions, the mentor repeatedly suggested to the student that he see the faculty psychologists, as it was clear that he was not feeling well. He fell asleep in class on several occasions. Even though the room temperature was comfortable, he refused to take off his coat and looked very pale.

It is very common for parallel adjustments, such as cultural etc. to place significant demands on students. They must not only get used to the culture of another country but also navigate the higher education system of that country, which is by no means an easy task and requires complex adaptation.

### 4.2 Presenting problem

When the student found the Student Support Centre, the question arose as to whether medical assistance was needed, but the student indicated that he had already visited his family doctor the day before and nothing specific had been diagnosed. He did not have a cold or any other physical illness according to a medical professional. The doctor recommended that the student get some rest.

At first, the student did indeed cite fatigue, saying he could not really sleep, that he thought about his family back home very often, and he was having a hard time finding his place in the university environment.

As for the client’s physical appearance, he is of medium height and thin built; he does indeed give the impression of someone who does not eat regularly.

### 4.3 Counselling process

Throughout our collaboration, I felt my role was becoming increasingly clear, so it was substantial to establish that the psychologist is bound by confidentiality and to clarify exactly how I could assist him. The first three sessions were dedicated to clarifying the role of a psychologist in a counselling setting.

At the end of those sessions, I felt he might not come back, but after the third session, that changed because he started to really stick to our schedule and began coming regularly; he wasn’t late and was punctual, even though he said it was hard for him to get out of bed. He arrived in Hungary in August because plane tickets were cheaper then, so until September he travelled to many places and got to know the country. He really wanted to go home for a few weeks during winter break, but his parents said they’d rather visit him, so he did not buy a plane ticket. Then it turned out his parents could not come after all, but by then it was too late to buy a ticket. He talked a lot with her parents and friends back home during the break. He felt good then, but starting in February, he began to feel less and less well.

### 4.4 Narrative development of the case

#### 4.4.1 Phase 1: disorientation and self-doubt

In the beginning, he had a lot of questions about why he was even at this university in this country and vocalizes his inner thoughts like this:

“I applied to a lot of places, but somehow, during the interviews, this university—and the fact that it’s a Hungarian university—really appealed to me.”

“Lately, I feel that it used to mean a lot to me that I was friends with the other students who came from my country. But lately, it’s as if I can’t even connect with them anymore; it’s as if no one understands me.”

#### 4.4.2 Phase 2: emerging awareness of cultural differences

“I often feel like I have two personalities. I haven’t gone crazy, but it really feels like one part of me is already a little ‘Hungarian’—I know how to behave and what the teachers and the dorm expect of me— but there’s another part of me that’s inconsolable because I’m so sad—I miss the flavours of home and the breeze back home.”

“Even when I cook something from home, it still tastes different, and until now that made me happy or at least comforted me, but it hasn’t gotten any better now.”

“I feel like I have two versions of myself. One that knows how to behave back home, and one that I am trying to build here—but they don’t match.”

#### 4.4.3 Phase 3: active adaptation and identity work

The client expressed that he needed to rediscover himself and see that he could carry himself in this new self. I praised him several times, noting that even though he had chosen a very difficult field of education, it was wonderful that he wasn’t giving up on his studies and wasn’t letting himself fail his exams.

In the Hungarian higher education system, a grade of 5 is the highest and a 1 means failure. He receives grades of 3 and 4. It was difficult for him to get used to not achieving the highest grade possible, as he had always been at the top of his class, but now he is no longer the top student in his class—yet he still achieves excellent results.

### 4.5 Result

As a counsellor, I made a real effort to keep the client grounded in the present moment and to reinforce the resources they already had at their disposal (e.g., family relationships, peer relationships (friends, roommates) or personal strength such as resilience). Cultural integration or adaptation is a very long process, and experiences of success and failure are ongoing. I felt like I needed to reassure them about how well they were progressing in their cultural adaptation and in finding their identity.

## 5 Psychological process 2: cognitive dissonance in cultural context

### 5.1 Description

The second case study examines individual issues that arose during a group process.

### 5.2 Group case study

We encountered various forms of cognitive dissonance in a group setting within the context of mentoring. Mentoring takes place during the students’ first university semester, after their arrival and enrolment, specifically within the first 12 weeks. Students reported interactions for which they sought help from mentors in interpreting. They wanted to move forward or correctly understand the situation by seeking support from their mentor.

One student reported that a fellow student openly argued against the instructor’s position:

“My classmate stated in the classroom that the professor might be wrong about a course material and then a very well-founded debate ensued. The instructor even praised the student for asking such good questions, and then the whole group just continued the discussion. It was shocking to see that the professor wasn’t upset about the interaction. As far as I was concerned, that was disrespectful of the student to contradict the teacher’s statement.”

This scene triggered cognitive dissonance, as the student’s belief system clashed with an observed behaviour. Specifically, the idea that respect implies the absence of confrontation, and that respect can also coexist with open disagreements. The student reported that he could not believe his ears, did not know how to make sense of the situation, and described minimal symptoms of anxiety because he did not fully understand the situation.

This is a classic double-bind situation, where the individual believes that both behavioural outcomes are associated with negative consequences (Bateson et al., 1956). This type of cognitive overload can lead to decision paralysis and dropout, as the client increasingly refrains from participating in academic discourse.

In another example, independent work and brainstorming were expected in a group setting, while the student was hoping for structured experiences and guidance:

“They just gave us an assignment and expected me to start working with others and share ideas. I felt very stuck. I was waiting for someone to provide structure because I didn’t even know where to start. I didn’t want to overstep my boundaries.”

This example also involves cognitive dissonance regarding roles. In the group dynamics roles contradict each other: one is expected to take on a leadership role, and the other has a different internal script about the role that contradicts a leadership role. The student feels a high degree of disorientation. Students perceive the instructions for the assigned task as a violation of norms, which may cause them to withdraw and reduce their motivation to study.

### 5.3 Coping strategies at the adaptive and maladaptive levels

#### 5.3.1 Rationalization

During group mentoring sessions, we strive to provide a framework for interpreting the situations described. And at this early stage in the first 3 weeks of group mentoring, rationalization emerged, which initially reduced the cognitive dissonance associated with the situation.

In this situation, rationalization did indeed reduce the dissonance, but it also led to a devaluation of the new cultural environment.

“How can I take the university work going on here seriously? I think it’s too lax and informal.; The instructor should act much more decisively.”

This strategy did indeed result in a temporary reduction in tension, but it also created an obstacle to adaptation because it rigidly maintained a sharp distinction between those inside and outside the group, which reinforced cultural polarization.

#### 5.3.2 Avoidance and cognitive suppression

This internal “explanation” can also be dangerous because it may reinforce situations that trigger cognitive dissonance (e.g., not speaking up in class or only talking to certain people). While this strategy may reduce cognitive dissonance in the short term, there is a risk that maintaining this explanation could lead to academic difficulties and social isolation, which in turn could increase stress.

#### 5.3.3 Adaptive attitude change and cognitive restructuring

When we were able to guide students in the group toward questions indicating guided cognitive restructuring, we succeeded in reinterpreting the situation and beliefs and creating a flexible and context-sensitive interpretation, which triggered a much more adaptive behavioural change.

“In class, asking questions is actually a sign of interest, and the instructor encourages these questions to help us delve deeper into the material. Asking questions previously seemed like questioning the professor and was seen as disrespectful”.

This is what we call accommodative schema modification, which modifies existing cognitive frameworks through the active integration of new cultural information. In the process of accommodative schema modification the learner does not abandon their previous system of values and norms, but rather reinterprets it within this context, leading to a form of situational differentiation.

#### 5.3.4 Integration and behavioural adaptation

The more definable the dissonance to the student is, the more tolerable and comprehensible the situation is to them. The student began to experiment with their behaviour, “testing” new interaction styles and awaiting the reactions of their environment. The positive feedback resulting from these experiences helped reinforce the new behaviour or response and reduced anticipatory anxiety:

“In the next small-group session, I tried to express my opinion … and I got the impression that this time my groupmates built on it and took my idea further. I was pleasantly surprised and proud that they had developed my idea further.”

This phase of the group mentoring increases the sense of self-efficacy and facilitates the internalization of new social norms, thereby reducing the emotional intensity of experiencing cognitive-emotional conflicts.

### 5.4 Result

By the end of the group mentoring process, the student experienced a significant reduction in distress symptoms related to dissonance and understood the essence of acquiring greater cognitive flexibility. Instead of experiencing the value conflict as a threat to self-coherence, they began to perceive it as a manageable and context-dependent phenomenon:

“I still feel the difference; it’s good to realize that I can accept others’ opinions—it enriches me rather than “threatening” me—but now I can choose how to react, depending on where I am and who I’m with.”

In this case, resolving cognitive dissonance neither eliminated conflicting beliefs, nor did the client escape the “threatening” situation. Rather, they succeeded in reorganizing them into a more complex, coherent cognitive-affective unified structure, which enabled the strengthening of metacognitive awareness and the adaptive emergence of integrative functioning in social and group situations, experiencing this as a personal sense of accomplishment as well.

## 6 Discussion

This article offers an in-depth, process-oriented perspective on intercultural adaptation in a European higher education context, highlighting how psychological adaptation unfolds as a renegotiation of identity in response to environmental influences where individuals are constantly required to grapple with their reactions to cognitive dissonance (Bethel et al., 2020; Lai et al., 2023). Adaptation is neither a linear nor a purely behavioural process, but rather a sustained endeavour to create meaning, shaped by the continuous reinterpretation of the self and the environment (Mitchell and Paras, 2018; Crisp, 2010).

This article contributes to research on student adaptation, as well as to research on migration and mobility, and enriches the field of intercultural psychology. The case study goes beyond traditional models of academic and social adaptation, focusing on the role of identity-forming processes and highlighting the importance of recognizing students’ individual experiences of adaptation. By integrating psychological, intercultural, and educational perspectives, it offers a comprehensive framework for addressing and mapping similar cases of adaptation. The cases discussed confirm that successful adaptation depends not only on coping with university requirements but also on the extent to which the individual is able to rethink their self-concept within the new cultural, institutional, and communicative environment—a process that should be understood as a dynamic process of meaning-making. The field of intercultural psychology is also relevant here, as most European higher education settings must provide an international adaptation platform for students, since many enter higher education systems as international students.

The article also contributes to broadening the scope of migration and mobility research by showing that adaptation can occur even in contexts of temporary mobility, for example, in the context of international higher education, where students spend an entire academic program in another country, do not seek long-term integration, yet still engage in a continuous process of identity negotiation.

### 6.1 The interaction between identity renegotiation and cognitive dissonance

One part of the case study sought to demonstrate how identity renegotiation takes place, while the other part illustrated the cultural adaptation of responses to cognitive dissonance. The client’s well-articulated early experiences illustrate how unresolved cognitive discrepancies—as described by the theory of cognitive dissonance—can destabilise previously coherent identity structures (Hoshino-Browne et al., 2005; Janík, 2017). Situations such as classroom participation or communication with authority figures triggered dissonance between internalised cultural schemas and the expectations perceived in the host environment. Behavioural elements previously learned and acquired were not helpful in the current cultural environment (Hall, 1976; Nisbett, 2003).

It is essential to note that individuals initially experience these dissonance experiences as personal inadequacy and react sensitively to them. It is relevant to provide an appropriate interpretive framework that helps ensure that culturally induced tension is not interpreted as a personal deficit. This is consistent with previous research suggesting that maladaptive attribution patterns can hinder intercultural adaptation (Genkova and Schreiber, 2025; Genkova et al., 2025).

Discussing and externalizing these internal conflicts during counselling made it possible to move toward a renegotiation of identity. The emergence of bicultural self-reflection (“two versions of myself”) illustrates that dissonance, when processed within a supportive context, can act as a catalyst for integrative identity development and does not lead to fragmentation later (Orbe, 2008; Karanikola and Panagiotopoulos, 2025). This facilitates the development of adaptive coping strategies in the individual (e.g., problem-focused coping, such as actively seeking solutions, planning steps or seeking social support). When they find themselves in a similarly difficult-to-interpret cultural environment, they can apply the learned strategy, interpret the situation, and draw the right conclusions to facilitate cultural adaptation (Nguyen et al., 2026).

### 6.2 Intercultural adaptation as contextualised meaning-making

The cases described emphasize the role of interpretive frameworks and processes in facilitating adaptation outcomes (Mitchell and Paras, 2018). The path described by the client suggests that the critical turning point lay not merely in acquiring new skills, but in reinterpreting ambiguous situations. For example, reframing the academic disagreement from “potential disrespect” to “expected commitment” fundamentally altered the emotional value of participation (Jade et al., 2026).

From this perspective, intercultural competence can be understood as a form of cognitive-emotional flexibility, which encompasses the ability to maintain multiple, potentially conflicting cultural frameworks simultaneously (Genkova and Schreiber, 2025; Genkova et al., 2025). The client’s subsequent statement (“Maybe I can be both”) reflects an emerging integrative stance, which in the literature indicates adaptive adjustment in multicultural contexts (Wang and Huang, 2026).

### 6.3 Implications for higher education counselling and student retention

The findings have direct implications for university psychological support services, particularly regarding student retention—a central issue in internationalised higher education systems (Baker and Siryk, 1984; Bethel et al., 2020).

First, the case highlights that students who experience adjustment difficulties as personal failures are at increased risk of dropping out, both academically and socially (Hofhuis et al., 2023). In this sense, psychological counselling can serve a preventive function by helping to reframe these experiences within a cultural context (Mitchell and Paras, 2018).

Second, the counselling process teaches the use of guided reflection to facilitate adaptive meaning-making. Rather than focusing exclusively on problem-solving or skill development, interventions that specifically target interpretive frameworks may be more effective in supporting long-term adaptation.

Third, integrating identity-centred work into counselling appears particularly relevant for students who are not yet able to navigate multiple cultural expectations. Supporting students in articulating the various aspects of the self and viewing them as a complex whole can reduce internal conflicts and enhance psychological resilience (Qumseya, 2018).

These insights are particularly relevant in STEM educational contexts, where implicit norms regarding communication, autonomy, and critical thinking may differ significantly from students’ prior educational experiences, as this type of educational environment requires these skills (Irrmann, 2005). As such, culturally sensitive counselling can contribute not only to mental well-being but also to academic success (Lai et al., 2023).

### 6.4 Contributions to intercultural psychology in a European context

While much of the existing literature on intercultural adaptation has emerged from an Anglo-Saxon context, this case study highlights the specific characteristics of the Central and Eastern European higher education environment (Genkova et al., 2025). Subtle differences in academic culture, communication styles, and institutional expectations can generate unique forms of dissonance that existing models do not fully capture (Hall, 1976; Nisbett, 2003).

By providing a micro-level analysis, this study contributes to a more nuanced understanding of the local experience of adaptation. It also highlights the need for context-sensitive research that takes into account the diversity of European educational environments (Crisp, 2010).

### 6.5 Limitations and future directions

As a single case study, the findings cannot be generalised. Rather, they provide analytical insight into the underlying processes. Future research could build on this work by examining similar processes across multiple cases, integrating longitudinal designs exploring the role of institutional factors in shaping adaptation trajectories.

In addition, quantitative research could examine the relationship between identity integration, resolution of dissonance, and retention outcomes in larger student populations.

## 7 Conclusion

Individual or group counselling sessions can significantly facilitate identity negotiation and the adaptive resolution of cognitive dissonance in individuals’ lives. Recognising and raising awareness of these processes can be key to promoting both psychological well-being and academic success among international students in Europe.

### 7.1 Theoretical contribution

This study contributes to the literature on intercultural adaptation by proposing a practical approach and, building on this experience, developing a process-oriented perspective that integrates cultural differences, cognitive dissonance, identity negotiation, and adaptation outcomes into a single conceptual framework. Our research emphasizes the central role of identity flexibility and adaptation during the transition to higher education. We conceptualize adaptation as a continuous, identity-based process in which individuals actively reinterpret themselves in response to changing environmental demands. The findings contribute to the field of intercultural psychology by demonstrating how communication patterns and cultural expectations can trigger cognitive dissonance, which in turn encourages individuals to negotiate their identities and undergo developmental change.

### 7.2 Practical contribution

The purpose of this article was to convey our experiences in a practical format to institutional leaders, mentors, instructors, and psychologists working in higher education. The cases presented demonstrate that adjustment difficulties should not be interpreted solely as academic or individual problems of the student; rather, it is always worthwhile to consider the context within the phases of ongoing identity development. Individual and group counselling can help students cope with cognitive dissonance, support identity development, and facilitate the coping strategies necessary for adaptation.

In addition, mentoring programs can create a significant safe space for self-reflection, helping students integrate diverse cultural experiences into a coherent self-image. If adaptation is recognised as a process of identity formation, professionals can develop more comprehensive interventions—tailored to the specific characteristics of their own institutions—that simultaneously support psychological well-being and academic success.

### 7.3 Policy implications

In recent years, higher education in Europe has become increasingly international; universities should adopt policies that recognise the need for intercultural adaptation and help students cope with it psychologically by supporting the development of their identities. Orientation programs, mentoring systems, psychological counselling services, and intercultural training initiatives should be designed not only to impart practical knowledge but also to foster students’ identity development and sense of belonging. It may be beneficial for institutions to introduce integrated support frameworks that address academic, social, and psychological adjustment simultaneously. Such measures can contribute to improved student retention, well-being, and academic performance, as well as to successful integration into higher education communities.

## Data Availability

The original contributions presented in the study are included in the article/Supplementary material, further inquiries can be directed to the corresponding author.
